# Lightweight mobile stick-type water-based triboelectric nanogenerator with amplified current for portable safety devices

**DOI:** 10.1080/14686996.2022.2030195

**Published:** 2022-02-17

**Authors:** Kyunghwan Cha, Jihoon Chung, Deokjae Heo, Myunghwan Song, Seh-Hoon Chung, Patrick T.J. Hwang, Dongseob Kim, Bonwook Koo, Jinkee Hong, Sangmin Lee

**Affiliations:** a School of Mechanical Engineering, Chung-ang University, Dongjak-gu, Seoul, Republic of Korea; b Department of Biomedical Engineering, The University of Alabama at Birmingham, Birmingham, AL, United States; c Safety System R&d Group, Korea Institute of Industrial Technology (Kitech), Yeongcheon-si, Republic of Korea; d Green and Sustainable Materials R&d Department, Korea Institute of Industrial Technology (Kitech), Cheonan-si, Republic of Korea; e Department of Chemical & Biomolecular Engineering, College of Engineering, Yonsei University, Seodaemun-gu, Seoul, Republic of Korea

**Keywords:** Energy harvesting, triboelectric nanogenerator, mechanical energy, portable device, 70 New topics, Others, 206 Energy conversion, transport, storage, recovery < 200 Applications

## Abstract

Due to its abundance, mechanical energy is a promising ambient energy source. Triboelectric nanogenerators (TENGs) represent an effective mechanical energy harvesting method based on the use of contact electrification. The existing liquid-based TENGs can operate robustly without surface damage; however, the output of these TENGs is considerably smaller than that of solid-based TENGs. Notably, liquid-based TENGs in which the liquid directly contacts the conductive material can produce an electrical current of more than few mA. However, the liquid reservoir must have an adequate volume, and sufficient space must be provided for the liquid to move for generating the electrical output. To ensure a compact and lightweight design and produce electrical output in the low input frequency range, we introduce a mobile stick-type water-based TENG (MSW-TENG). The proposed MSW-TENG can generate an open-circuit voltage and closed-circuit current of up to 710 V and 2.9 mA, respectively, and be utilized as self-powered safety device. The findings of this study can promote the implementation of TENGs in everyday applications.

## Graphical Abstract

**Figure:**
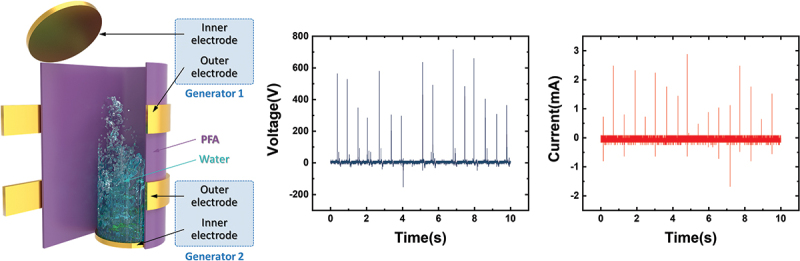

## 1. Introduction

With the increasing interest in internet of things (IoT) and small electronics, the demand for portable energy sources for power circuits and sensors has emerged. To power these devices, energy harvesting techniques have been developed to generate electrical power from the external environment of the generators. Among various ambient energy sources, such as solar and thermal energy, mechanical energy is a promising energy source owing to its abundance [1–3]. To effectively harvest mechanical energy, triboelectric nanogenerators (TENGs) have been developed, which can generate electricity through contact electrification [4,5]. Although TENGs have successfully powered portable electronics [6,7] and sensors for IoT applications [8–10] through mechanical motion, frictional damage inevitably occurs on the solid surface owing to the contact between two triboelectric materials [11,12]. To decrease this friction, the use of liquids as triboelectric materials was recommended [13–15]. Although liquid-based TENGs can operate robustly without surface damage, the generated output is considerably lower than that of solid-based TENGs. To enhance this electrical output, certain researchers developed liquid-based TENGs in which the liquid directly contacted the conductive material [16–18]. Through the continuous contact–separation between water and the electrode, the generator could produce an electrical current of more than few mA. However, the liquid reservoir was required to have a certain volume, and a certain amount of space was required for the liquid to move for generating the electrical output. This framework reduced the overall efficiency and increased the total size of the generator. To realize portable applications, it is necessary to adopt a liquid-based TENG that is compact and can produce a high electrical output with a limited mechanical input.

Considering these aspects, we developed a lightweight mobile stick-type water-based triboelectric nanogenerator (MSW-TENG) that can produce electrical output through mechanical motion applied to the device. As the water inside the MSW-TENG directly contacts the electrode, a high electrical output can be generated through the charge separation and accumulation induced by the self-ionization of water. For an input of 1.5 Hz, a single MSW-TENG could generate an open-circuit voltage (*V_OC_*) and closed-circuit current (*I_CC_*) of up to 710 V and 2.9 mA, respectively. As the size of the generator and amount of liquid considerably influence the portability and output production of the device, quantitative analyses were performed considering the size ratio of the electrode, physical space between the electrodes, and amount of water to determine the optimized device design. The proposed TENG could be utilized as a traffic safety light baton that can power 100 LEDs each time an operator manually shakes the baton. Notably, the proposed device can be used as a self-powered safety device, which widens the potential for implementing TENGs in everyday applications.

## 2. Methods

### 2.1. Fabrication of MSW-TENG

**Figure 1.**
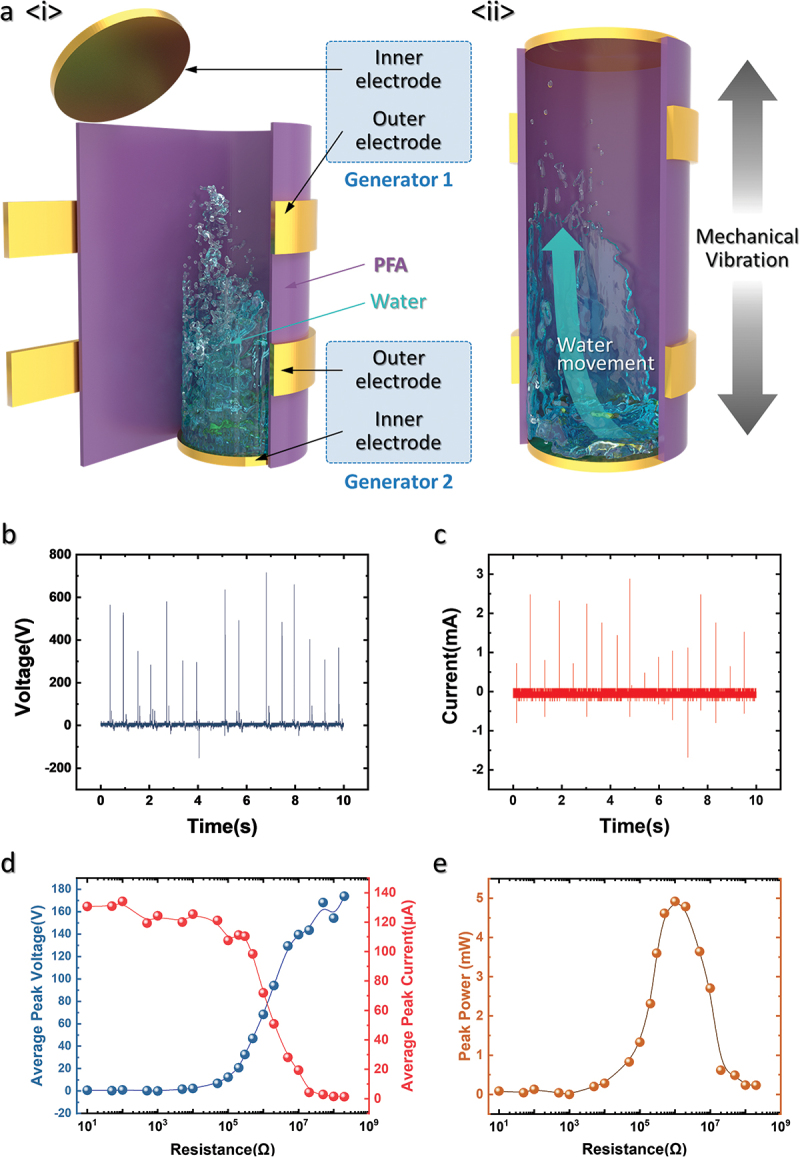
Mobile stick-type water-based triboelectric nanogenerator (MSW-TENG). (a) Schematic of MSW-TENG. (b) *V_OC_* and (c) *I_CC_* outputs of MSW-TENG. (d) Average peak voltage, current, and (e) power of MSW-TENG depending on the external load.

**Figure 2.**
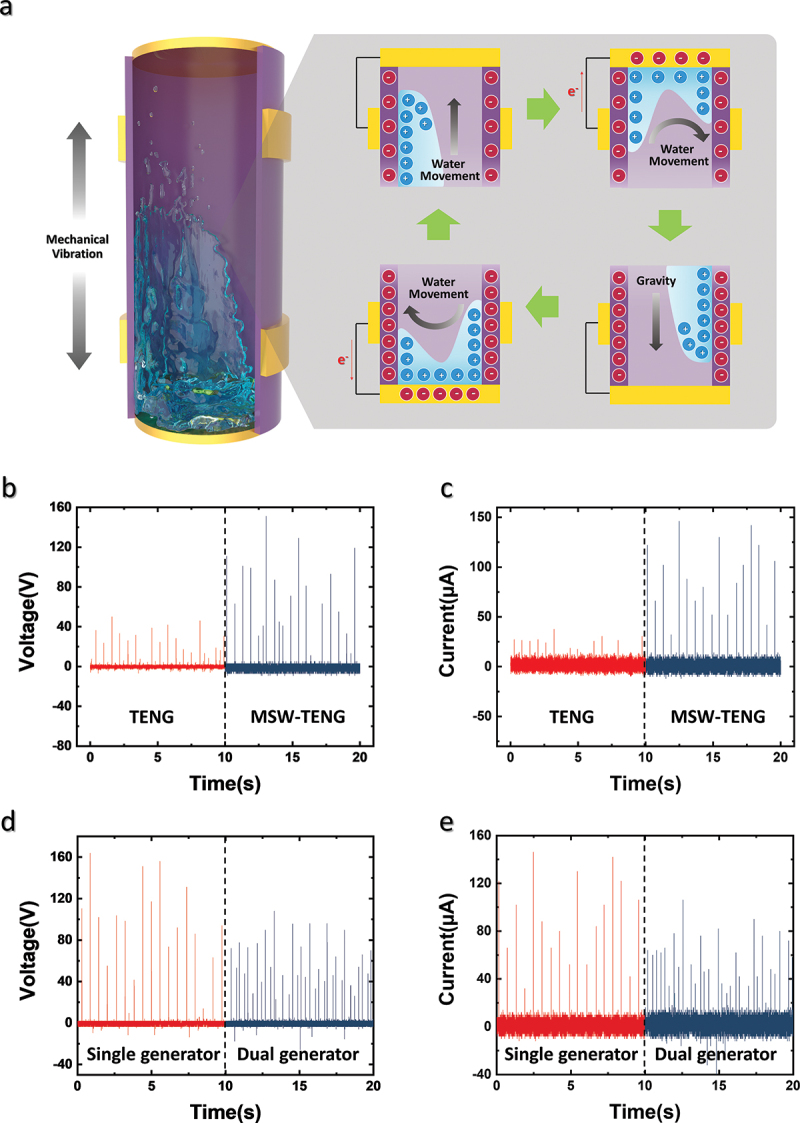
**Working mechanism and output of MSW-TENG**. (a) Working mechanism of MSW-TENG. (b) *V_OC_* and (c) *I_CC_* outputs for conventional TENG and MSW-TENG. (d) *V_OC_* and (e) *I_CC_* outputs of single- and dual-generator MSW-TENG.

**Figure 3.**
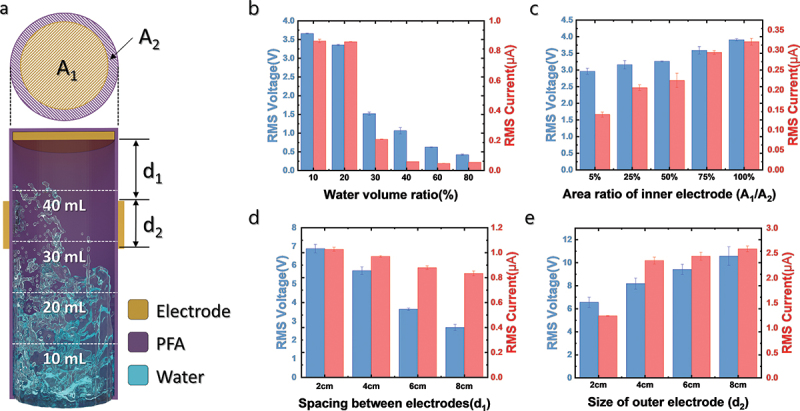
**Electrical output of MSW-TENG depending on various design parameters**. (a) Schematic of MSW-TENG. *V_RMS_* and *I_RMS_* outputs depending on the (b) water volume ratio, (c) area ratio of the inner electrode, (d) spacing between electrodes, and (e) size of the outer electrode.

**Figure 4.**
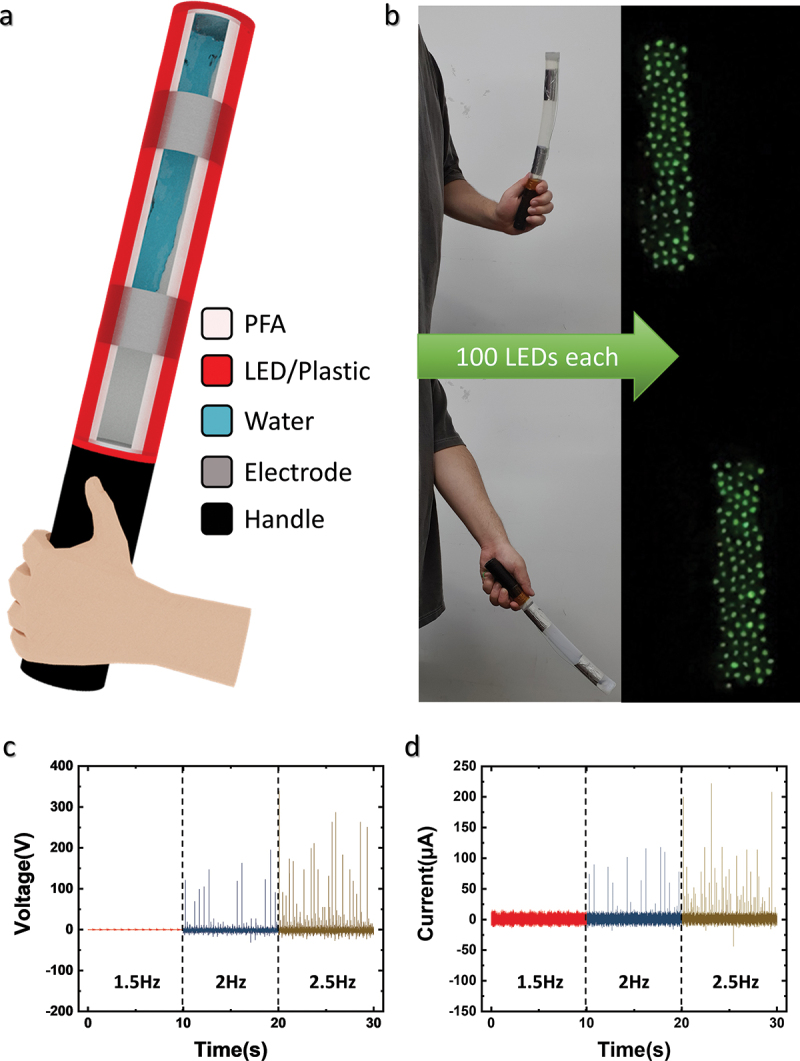
**MSW-TENG as traffic safety light baton**. (a) Schematic of MSW-TENG-based traffic safety light baton. (b) Photograph of baton powering a 100 LED array when manually shaken. (c) *V_OC_* and (d) *I_CC_* of MSW-TENG depending on the mechanical vibration frequency.

A 1-in perfluoroalkoxyalkane (PFA) hose (1.5 mm thick, HIFLON Co., South Korea) and DI water were used in the experiments. Except in the case shown in Figure 4, the length of the stick was 12.5 cm, and 10 mL of water was used (in the cases shown in Figure 1b, 1c, 4a, and 4b, the hose length was 25 cm). The inner electrode was a piece of aluminum tape (thickness of 0.05 mm, DUCKSUNG Co., South Korea), cut to match the inner diameter of the hose (22.4 mm). The same type of aluminum tape with a width of 2.5 cm was used as the outer electrode (however, in the cases shown in Figure 1b, 1c, and 4, the width was 5 cm) and attached 1 cm from the lid. To seal the hose lid and attach the inner electrode, a piece of polytetrafluoroethylene (PTFE) tape (50 mm wide and 0.08 mm thick, Chukoh Chemical Industries Co., Japan) was used. In the cases shown in Figure 2b and c, the inner electrode was covered with this tape to measure the output of the conventional triboelectric device. In the parametric study (Figure 3), the following settings were used: the stick contained 5, 10, 15, 20, 30, and 40 mL of deionized (DI) water (Figure 3b); the diameters of the inner electrode were 22.4, 20, 16, 11, and 5 cm (Figure 3c); the outer electrode was attached 2, 4, 6, and 8 cm from the inner electrode, i.e. lid of the stick (Figure 3d); and the electrode widths were 2, 4, 6, and 8 cm (Figure 3e).

### 2.2. Measurement and mechanical input

The electrical measurements, specifically the voltage and current measurements, were obtained using a mixed domain oscilloscope (MDO 3014, Tektronix Co., USA) and a low-noise current preamplifier (SR570, Stanford Research Systems Co.), respectively. The vertical mechanical movement was produced using a shaker including a motor (Motor: GBM-02JSK11, GR Electronics, South Korea; Gear head: S9KC10BH, SPG Co., South Korea) with an amplitude of 26 cm. The frequency of the shaker was adjusted (1 Hz/60 RPM) using a motor driver (BX-3000A, GR electronics, South Korea), and a frequency of 1.5 Hz (90 RPM) was primarily applied.

## 3. Results and discussion

Figure 1a shows the schematic of the MSW-TENG. As shown in Figure 1a-i, the MSW-TENG consists of a PFA cylinder that serves as both the substrate and triboelectric material, with the inner and outer electrodes placed on the top and bottom of the cylinder. A certain amount of DI water is filled in the tube to ensure consistent contact and separation when a mechanical input is provided. The two inner electrodes seal both ends of the PFA tube, and two outer electrodes are attached on the sides of the device. Each generator includes an internal and external electrode and a closed circuit forming a freestanding-type TENG [19]. As shown in Figure 1a-ii, water inside the cylinder sequentially contacts both the top and bottom inner electrodes as mechanical vibration is applied. Because the PFA has a negative surface charge [20,21] and water can undergo self-ionization, the water ions are affected by the electrical field, causing charge separation and accumulation to occur [22]. When the charge-separated water contacts the electrode, a high electrical output is generated.

Figure 1b and c show the *V_OC_* and *I_CC_* outputs of the MSW-TENG provided with mechanical vibration of 1.5 Hz, respectively. The maximum $V_{oc}$ and $C_{cc}$ are 710 V and 2.9 mA, respectively, and a positive peak-shape output is observed because the positively charged particles lead to a high output, as illustrated in Figure 2 and discussed in the subsequent text. Figure 1d and e show the average peak voltage and current under different external load resistance values, respectively. The MSW-TENG generates a power of approximately 5 mW when the device is vibrated at 1.5 Hz with a load resistance of 1 MΩ.

Figure 2a schematically illustrates the working mechanism of the MSW-TENG. When the MSW-TENG is vertically excited via the mechanical input, water exhibits sloshing motion in the container [23,24]. Owing to the self-ionizing nature of water, charge separation and accumulation occur when an external electric field is introduced. As mentioned previously, the container is made of PFA, which is a negatively charged material. The electric field from the PFA surface can separate and accumulate the hydrogen, hydronium, and hydroxide ions in water. As shown in Figure 2a, a positive charge is accumulated as the water rises toward the top electrode. When the water with the accumulated charge contacts the top electrode, a high output peak is generated as the electrons flow to the top electrode to achieve charge equivalence. After this contact, the water flows downward owing to gravity, and the electrons move toward the outer electrode. Owing to the relatively low speed associated with water separation and the presence of residue water on the electrode surface, the output is lower than that when water contacts the electrode. As the water flows downward, charge is again accumulated owing to the effect of the PFA surface. When the water with accumulated charge contacts the bottom electrode, a high electrical peak is generated, and the generation process is restarted. This process occurs repetitively, and the MSW-TENG produces an alternative current output when external mechanical vibrations are continuously applied.

Figure 2b and c, respectively, show the output *V_OC_* and *I_CC_* values of conventional TENG and MSW-TENG to facilitate a comparative analysis. As shown in Supplementary Material 1, the conventional TENG has the same structure and design parameters as those of the MSW-TENG, but the top and bottom electrodes are not directly exposed to water. The average peak voltage and current of the MSW-TENG are more than two times those of the conventional TENG because water with accumulated charge directly contacts the conductive material.

Figure 2d and e, respectively, show the *V_OC_* and *I_CC_* values for an MSW-TENG with a single generator on the top and dual generator on both the top and bottom to facilitate a comparative analysis. In the single generator framework, the end of the tube to which electrodes are not attached is covered with a lid (in this study, the end was sealed using a PTFE tape). The peak output of the MSW-TENG with a single generator is higher than that of the dual generator; however, the dual-generator produces more output peaks compared to the single generator.

Figure 3a shows the schematic of the MSW-TENG with various design parameters. *A_1_* and *A_2_* represent the area of the inner electrode and internal diameter of the cylinder, respectively. *d_1_* is the distance between the two electrodes, and *d_2_* is the length of the outer electrode. To compare the electrical output associated with different design parameters, the root-mean-square (RMS) values were calculated to evaluate the continuous power as the MSW-TENG generated a sharp peak-type output. The RMS voltage (*V_RMS_*) and current (*I_RMS_*) were calculated as follows:

$V_{rms}=\sqrt{\int^{\frac{V{\left(t\right)}^{2}dt}{T}}}$, $I_{rms}=\sqrt{\int^{\frac{I{\left(t\right)}^{2}dt}{T}}}$

Figure 3b shows the *V_RMS_* and *I_RMS_* values of the MSW-TENG with different water–PFA cylinder volume ratios. The total volume of the PFA cylinder is approximately 50 mL (cylinder diameter of 0.23 cm and height of 12 cm); 5 mL and 40 mL of DI water are used, occupying 10% and 80% of the volume of the device, respectively. As shown in Figure 3b, the water volume ratio of 10% generates the highest output, and the output declines as the water volume ratio increases. This finding indicates that as the water volume increases, the space for charge accumulation becomes limited. As mentioned in the previous paragraph, the main working mechanism for high output generation is charge separation and accumulation in water owing to the electric field of the PFA surface. As the empty space is occupied by water, less space is available for water to move and possess concentrated charge. In addition, as the PFA cylinder is filled, water naturally screens the electric charge on the PFA surface by creating an electrical double layer (EDL) [25–27]. Owing to the low-intensity electric field on the PFA surface, the MSW-TENG produces a lower output when most of the cylinder is filled with water. The *V_OC_* output plot against time for different volume ratios of water is shown in Supplementary Material 2.

Figure 3c shows the *V_RMS_* and *I_RMS_* values for different sizes of the inner electrode. The inner diameter of the PFA cylinder is 23 mm, and the area is 415.265 mm^2^. The electrode area ratios of 5%, 25%, 50%, 75%, and 100% in Figure 3c correspond to inner electrode diameters of 5, 11, 16, 20, and 23 mm, respectively. Both *V_RMS_* and *I_RMS_* increase as the electrode area ratio increases. When water collides with the top surface of the PFA cylinder, mechanical energy is lost, and the velocity of water significantly decreases. In general, the charge separation and accumulation of water are closely related to the velocity of water [18]. Therefore, the water that contacts the electrode after having been in contact with the dielectric material is expected to have a less accumulated positive charge. As the size of the inner electrode reduces, the probability of water with a high mechanical energy contacting the inner electrode decreases, and a lower electrical output is generated. The *V_OC_* plot against time for different sizes of the inner electrode is shown in Supplementary Material 3.

Figure 3d shows the *V_RMS_* and *I_RMS_* values of the MSW-TENG for different distances between the inner and outer electrodes. A 2-cm-wide electrode is attached on the side of the PFA cylinder at distances of 2, 4, 6, and 8 cm from the top surface of the cylinder. The MSW-TENG produces a higher electrical output when the electrode is placed closer to the top surface of the cylinder. When mechanical vibration excites the water, the water inside the cylinder forms a triangular shape owing to gravity [18]. Consequently, a larger area of the external electrode is covered by water as the outer electrode is close to the bottom of the cylinder. As the water screens the electrical potential of the PFA, it produces less electrical output. The *V_OC_* plot against time with different distances between the inner and outer electrodes is shown in Supplementary Material 4.

When the device has a wide outer electrode, additional space is available to supplement the area covered by the water. Figure 3e shows the *V_RMS_* and *I_RMS_* outputs depending on the length of the outer electrode. Both *V_RMS_* and *I_RMS_* increase as the length of the outer electrode increases because more area is exposed to the air, yielding a large potential difference. The *V_OC_* plot for different outer electrode size is shown in Supplementary Material 5. In addition, the length of the PFA cylinder is also a key parameter in fabricating the MSW-TENG because long cylinder can makes more charge separated inside water. As shown in Supplementary Material 6, the electrical output increases as the length of the PFA cylinder increases.

The proposed MSW-TENG can produce a high electrical output with a low input frequency of 1 to 3 Hz and is lightweight because only 10 mL of water is used. Therefore, the proposed device can be used in various portable applications. Figure 4a schematically illustrates an MSW-TENG-based safety traffic light baton. The PFA cylinder containing water and the electrodes is covered by red plastic. As shown in Supplementary Material 7, two TENGs are installed at the top and bottom of the cylinder to produce a higher electrical output with a single input. As shown in Figure 4b and Supplementary Videos 1 and 2, a 100-LED array connected to each generator can be powered when water contacts the electrode each time. Even in the low-frequency range, as the water lightly contacts or falls from the top and bottom of the cylinder, all the LEDs are lit. Figure 4c and d show the output *V_OC_* and *I_CC_* depending on the input frequency. The electrical output of the MSW-TENG is measured using a mechanical vibration device, as shown in Supplementary Material 8. The output increases with the increase in the input frequency because the higher mechanical input leads to high-velocity water movement, which increases speed and the number of times that the water contacts the electrode.

## 4. Conclusion

We developed a compact and lightweight MSW-TENG that can generate a high electrical output while operating in the low input frequency range. Owing to the charge accumulation and separation induced by the charge on the PFA surface, the proposed device can generate a high electrical output each time the water contacts the electrode inside the cylinder. The electrical output of the MSW-TENG is influenced by various design parameters such as the amount of liquid inside the device, area of the top electrode, spacing between the two electrodes, and length of the outer electrode. The highest output is generated when the MSW-TENG has adequate space for water to move and a high electrode area. The proposed MSW-TENG can generate an electrical output of up to 710 V and 2.9 mA with a mechanical input of 1.5 Hz. The MSW-TENG can be used as a safety traffic light baton that can power an LED array when manually shaken.

## Supplementary Material

Supplemental Materials

Supplemental video 2

Supplemental video 1
