# Supplementary material for: Lightweight mobile stick-type water-based triboelectric nanogenerator with amplified current for portable safety devices
Source: Sci Technol Adv Mater. 2022 Feb 17;23(1):161–8. doi: 10.1080/14686996.2022.2030195 (PMC8856095; doi:10.1080/14686996.2022.2030195)
Supplement: Supplemental Materials [file TSTA_A_2030195_SM0317.docx]

**Supplementary Materials**


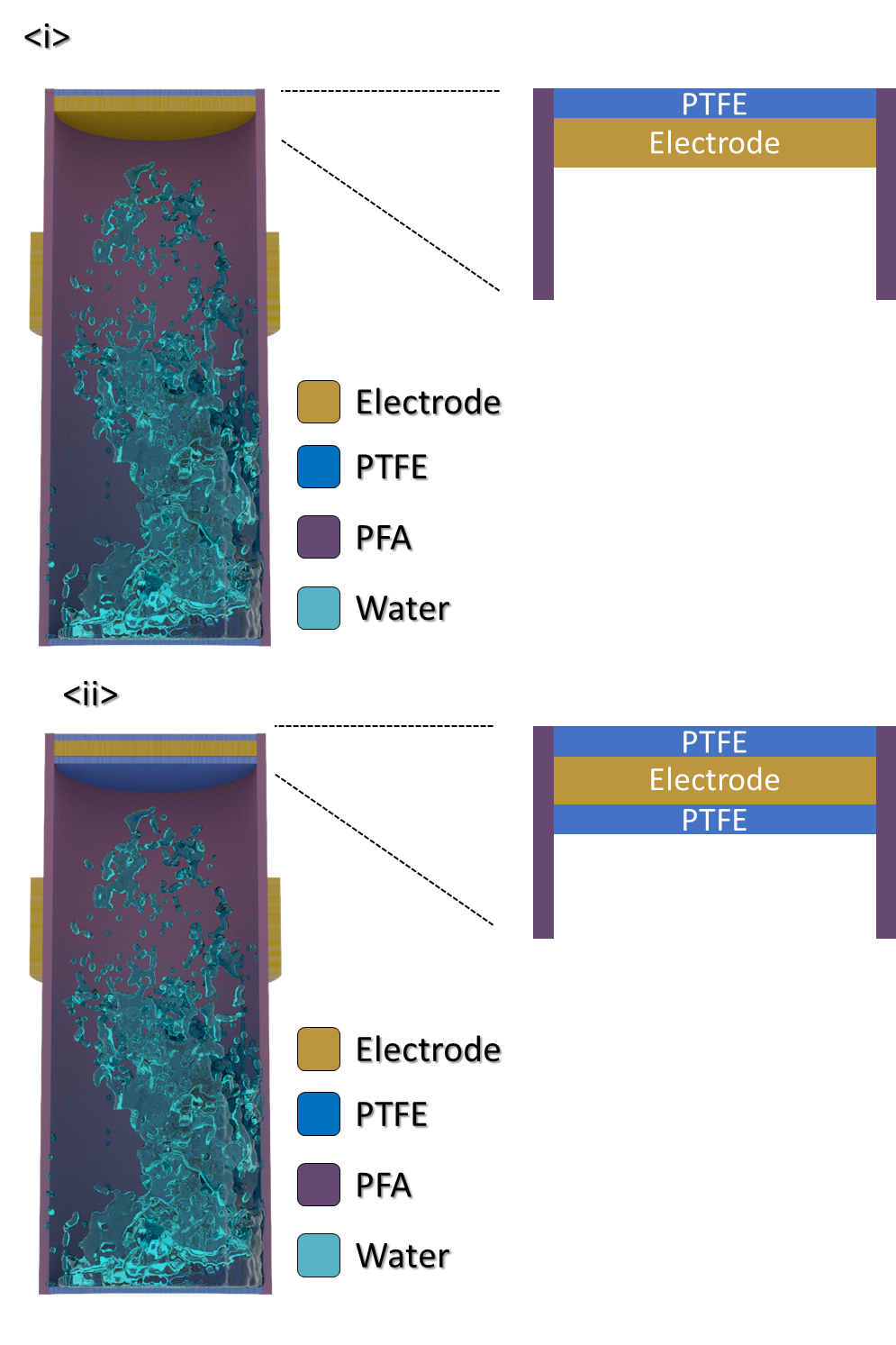


Supplementary Information 1| The structure of <i> conventional TENG and <ii> MSW-TENG.


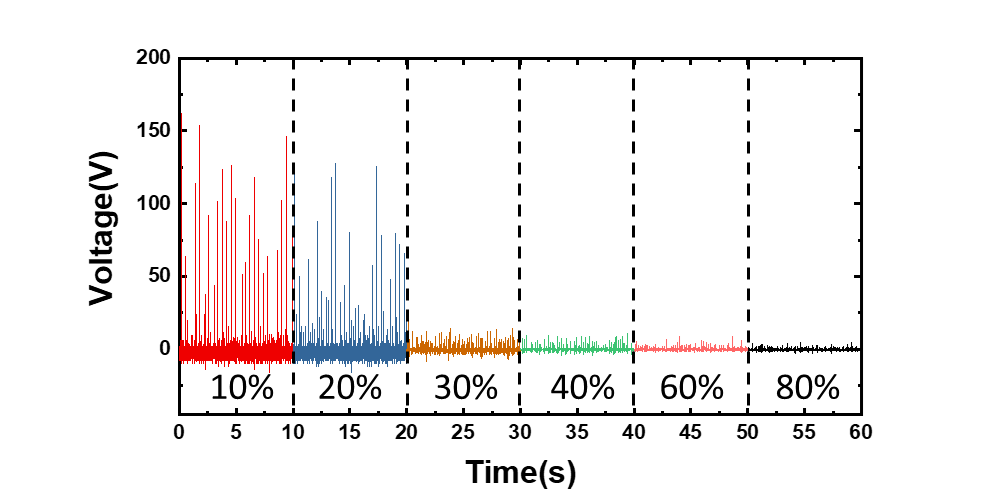


Supplementary Information 2| Open-circuit voltage (*V_OC_*) output plot against time for different volume ratios of water.


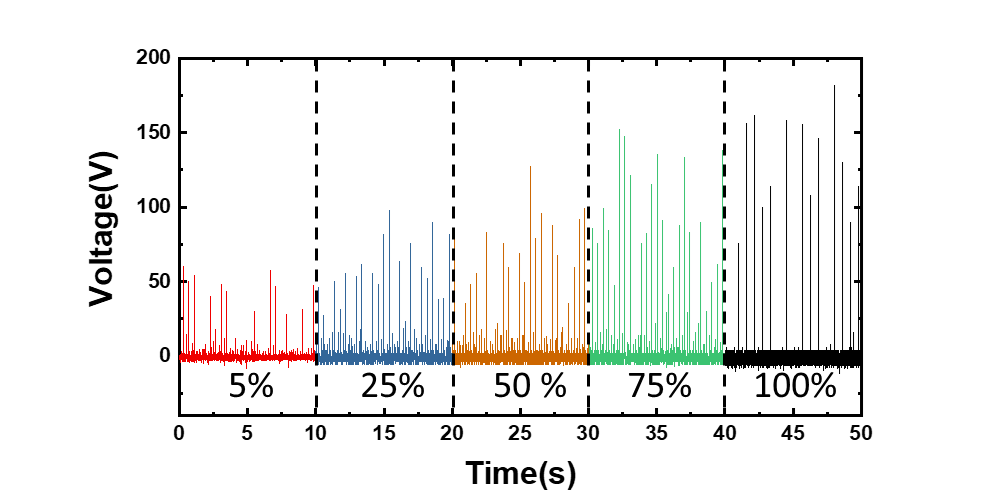


Supplementary Information 3| The V_OC_ plot against time for different sizes of the inner electrode. The inner diameter of PFA cylinder is 23 mm and the area is 415.265 mm^2^. The electrode area ratios of 5%, 25%, 50%, 75% and 100% in supplementary information 3 correspond to inner electrode diameters of 5, 11, 16, 20, and 23mm. The single-generator device has 12cm of height, 10ml of DI water and the 2cm wide outer electrode placed 2cm far from inner electrode.


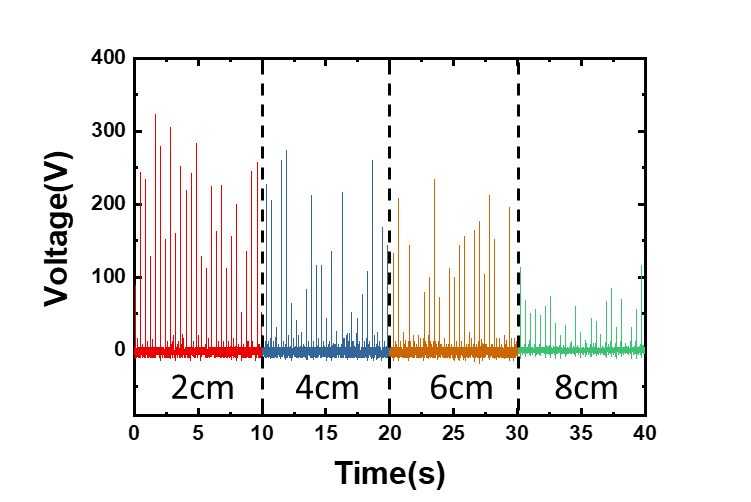


Supplementary Information 4| The *V_OC_* plot against time with different distances between the inner and outer electrodes.


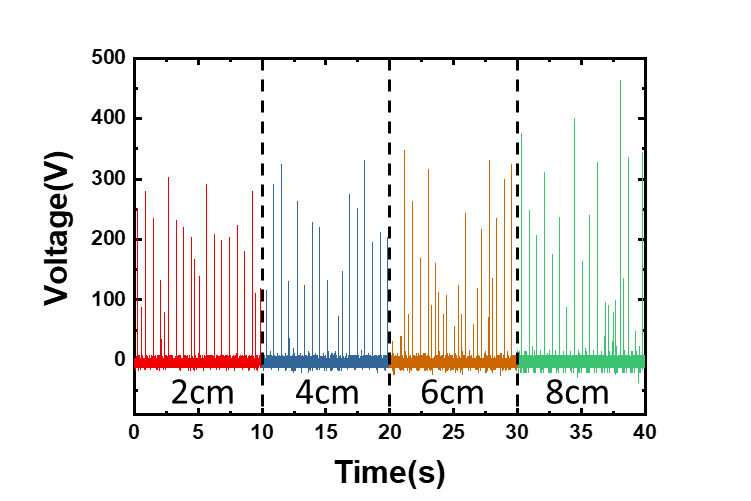


Supplementary Information 5| The *V_OC_* plot against time for different outer electrode size.


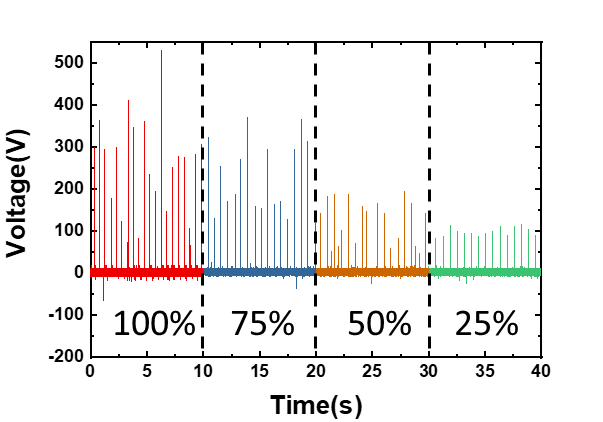


Supplementary Information 6 | The *V_OC_* plot against time with different height of PFA cylinder.


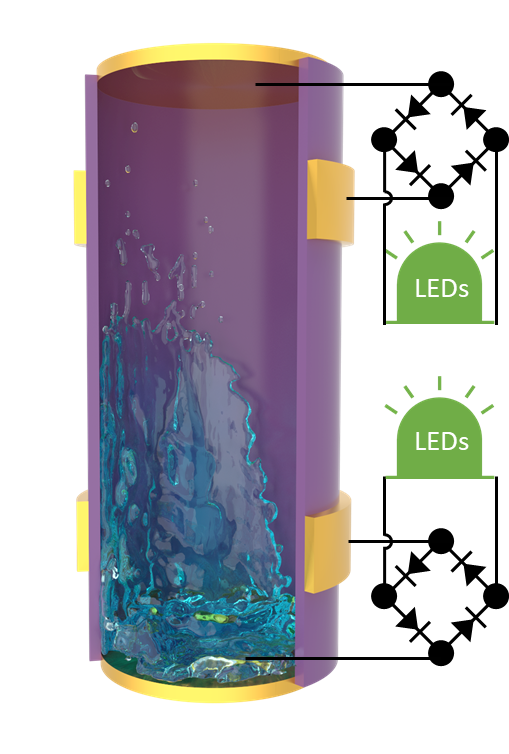


Supplementary Information 7| The diagram of MWS-TENG and circuit.


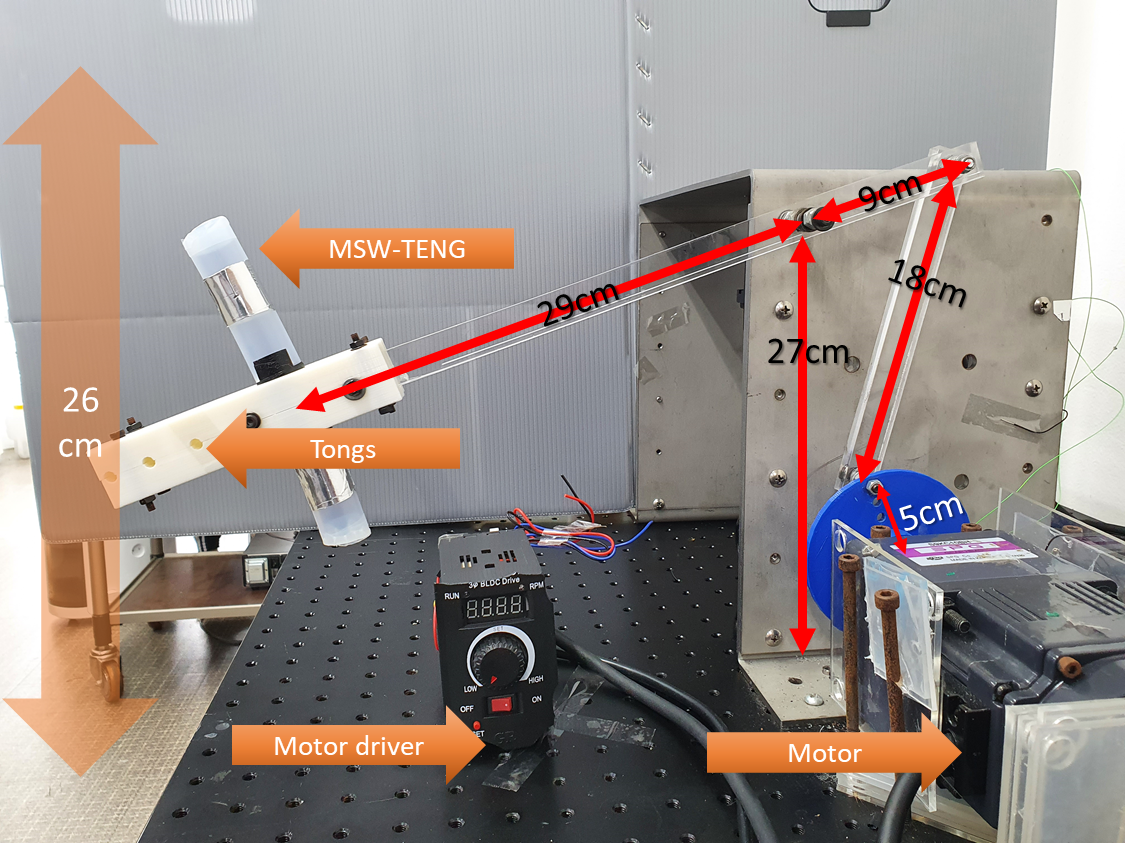


Supplementary Information 8| The design and dimension of stick shaker. This device has 26cm amplitude and frequency of the shaker was adjusted (1HZ/60RPM) using a motor driver. MSW-TENG are caught by PLA Tongs, made by 3d printer(2X, Sindoh Co.)

Supplementary Movie 1.

Video for MSW-TENG-based safety traffic light baton in rotational motion

Supplementary Movie 2.

Video for MSW-TENG-based safety traffic light baton in running motion
